# Accelerating the Gillespie *τ*-Leaping Method Using Graphics Processing Units

**DOI:** 10.1371/journal.pone.0037370

**Published:** 2012-06-08

**Authors:** Ivan Komarov, Roshan M. D’Souza, Jose-Juan Tapia

**Affiliations:** 1 Department of Mechanical Engineering, Complex Systems Simulation Lab, University of Wisconsin-Milwaukee, Milwaukee, Wisconsin, United States of America; 2 Department of Computational Biology, University of Pittsburgh, Pittsburgh, Pennsylvania, United States of America; German Cancer Research Center, Germany

## Abstract

The Gillespie *τ*-Leaping Method is an approximate algorithm that is faster than the exact Direct Method (DM) due to the progression of the simulation with larger time steps. However, the procedure to compute the time leap *τ* is quite expensive. In this paper, we explore the acceleration of the *τ*-Leaping Method using Graphics Processing Unit (GPUs) for ultra-large networks (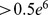 reaction channels). We have developed data structures and algorithms that take advantage of the unique hardware architecture and available libraries. Our results show that we obtain a performance gain of over 60x when compared with the best conventional implementations.

## Introduction

The Gillespie Stochastic Simulation Algorithm (GSSA) [1] and its variants [2], [3] are cornerstone algorithms for stochastic simulation of chemical kinetics with very important applications in modeling a variety of biological phenomena. The GSSA is applicable where the small number of reactant molecules in the system does not allow deterministic modeling using coupled ordinary differential equations. The GSSA is essentially a random walk over the set of reaction channels and exactly represents the distribution of the chemical master equation [4].

The original formulation of the GSSA, called the Direct Method (DM) [1], is prohibitively expensive to compute as it advances the simulation one reaction at a time. Much work has been done to improve the computational complexity. The next reaction method [3] and Optimized Direct Method(ODM) [5] improve performance by reducing redundant reaction propensity calculation by using dependency graphs. Additionally, various heuristics have been used to reduce the complexity of finding the next reaction to be fired [6]–[8]. All these methods are exact solutions.

The second approach to accelerating GSSAs is through approximation, where several reaction-channels are simultaneously processed within a given update step under the assumption of mutual independence in the computed time advancement. The first effort in this direction was the 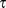-Leaping Method [2]. Several modifications to the original 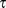-Leaping Method address various optimization and correctness issues [9]–[12]. The ability to advance the system by firing multiple reactions in a given update step significantly reduces overall simulation time.

The third approach to accelerating GSSAs is through parallelization. Coarse-grain parallelization, where several independent runs of a given system are executed in parallel to generate statistically dense data-sets, has been implemented on CPU clusters [13], multi-core CPUs [14], and Graphics Processing Units (GPUs) [15]. These efforts are limited by the fact that large networks still take an inordinate time to compute. Fine-grained parallelization efforts accelerate the simulation of a single run. This type of parallelism is more complex due to synchronization and communication issues. Such efforts have included newer parallel hardware such as GPUs [16] and Field Programmable Gate Arrays (FPGAs) [17]. The latter platform is inflexible due to the level of programming complexity. Moreover, due to the limited hardware resources, it cannot handle systems with greater than 10^4^ reaction channels.

Our work differs fundamentally from the first set of parallelization efforts because we are concerned with fine grained parallelization. To the best of our knowledge, no other fine-grained parallelizations of the 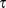-Leaping Method have been reported in the literature. Thus our work is quite different from, and cannot be directly compared with, other fine grained parallelization efforts.

## Results

We evaluated the performance of our system against StochKit [18], a suite of efficient serial GSSA implementations. StockKit was compiled with gcc4.4 with the appropriate optimization flags and executed on Intel i7 930 with 6GB of RAM. The operating system is Windows 7. Our parallel GPU code was run on a consumer grade NVIDIA 480GTX GPU (Fermi architecture).

**Figure 1:**
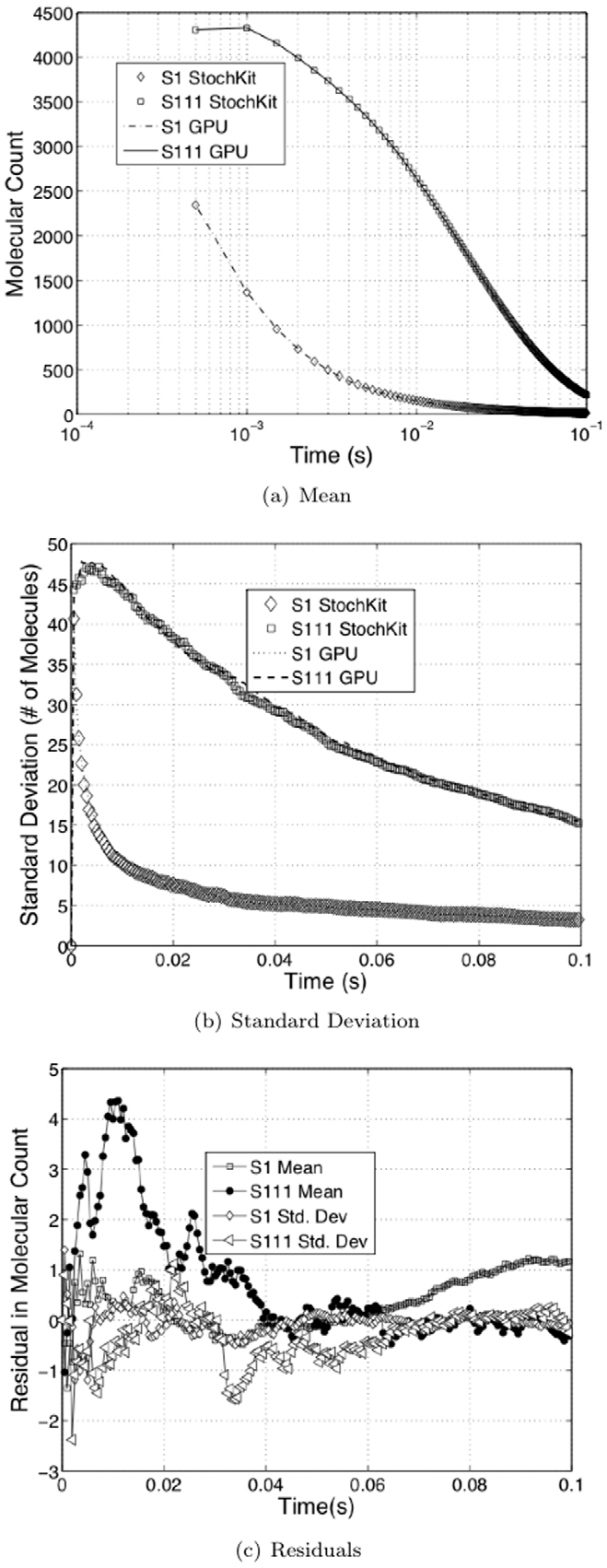
Output comparison with StochKit for accuracy. A random synthetic network with 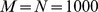 was used. Two species, namely, 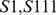 were tracked and compared with StochKit output. Figure 1a shows the comparison of means. Figure 1b shows the comparison of standard deviations. Figure 1c shows the residuals between StochKit outputs and GPU outputs. The maximum deviation on the means is about 0.16%. The maximum deviation on standard deviations is about 4%.

We used an in-house script capable of generating consistent large synthetic networks (Appendix S1) to test our system. These synthetic networks are square, i.e., the number of reactions *N* is equal to the number of reactant species *M*. For accuracy tests, we created a random synthetic network where 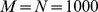. We chose to track the time trajectories of two reactants, namely, 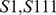, and compare them with trajectories obtained from StochKit. We ran 1000 runs to collect the data. Figure 1 shows the results. The maximum deviation on the means is about 0.16% and the maximum deviation on the standard deviations is about 4%.

**Figure 2:**
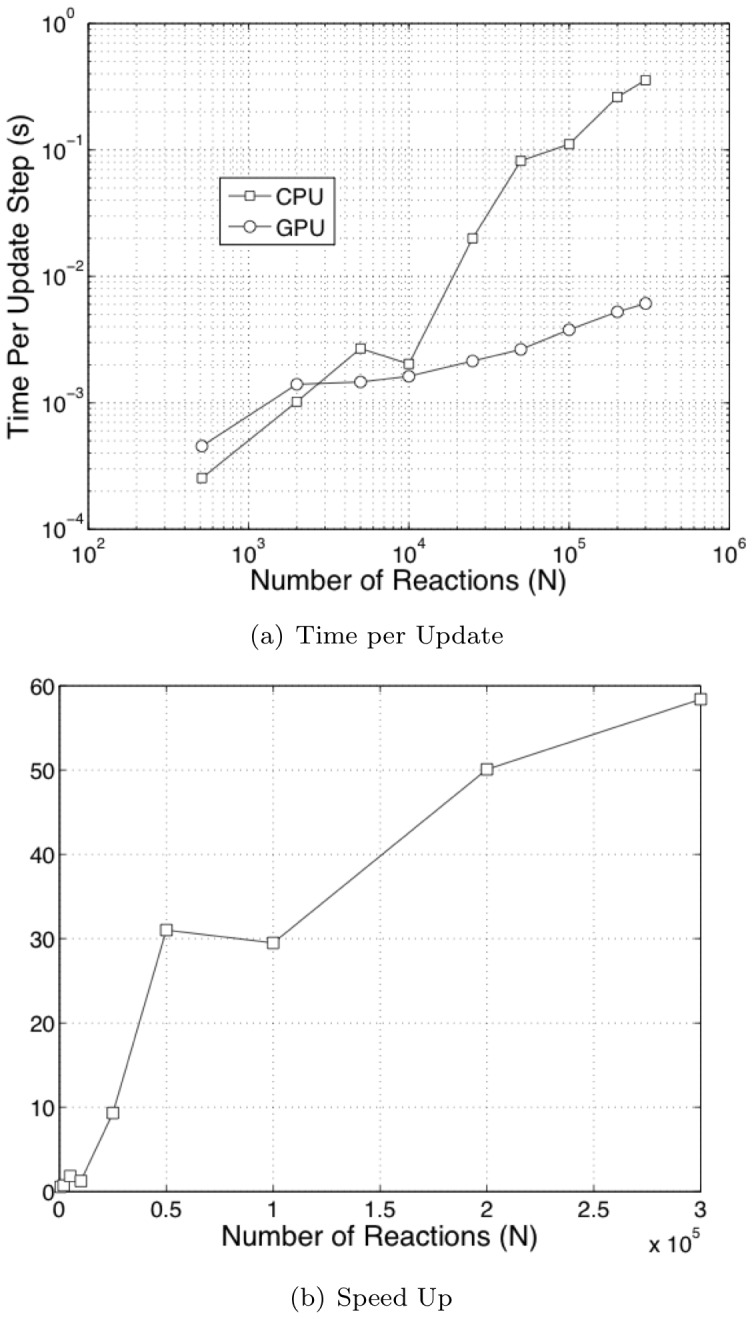
Performance comparison with StochKit.

**Figure 3:**
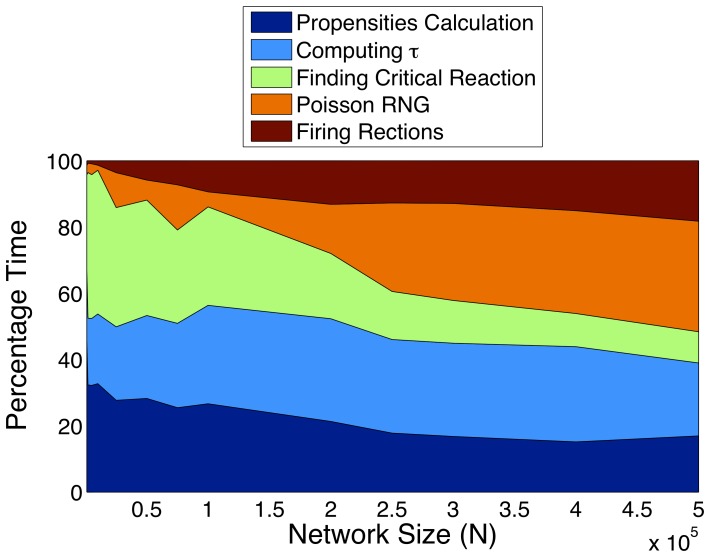
Relative computation times of various kernels.

We checked the performance of our parallelized implementation against StochKit. Figure 2 shows the performance benchmarks vs. StochKit. The break-even point between the serial CPU version and GPU version is about 10^3^ reaction channels. For smaller systems, the computational resources on the GPU are underutilized. The best speed-up we obtained was 60x where the number of reactions was on the order of 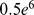. The benchmark time in both cases only involves computation of the actual algorithm and not the problem set-up. In the case of StochKit, the problem set-up phase is very slow and we observed end-to-end speed-up of over 600x for systems with 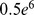. Finally, we analyzed relative computation times of various kernels in our GPU implementation. Figure 3 shows the results for varying problem sizes. It can be seen that for large systems, the 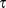 leap calculation and poisson random number calculation dominates. For smaller systems, when the GPU is underutilized, the dominant kernels are the ones for computing propensities and finding the critical reaction. The Intel i7 930 core is rated at about 30 GFlops per core for single precision. The NVIDIA 480GTX is rated at 1.5 TFlops, i.e., a 50× advantage in raw computing power. At 60× gain in performance it is evident that our implementation performs better than what the raw computing power advantage of the GPU would suggest. While our memory access patterns may not be ideal because of the stochastic nature of the algorithm, we surmise is StochKit has the same exact problem. However, the enhanced bandwidth of the GPU gives us the extra edge in performance.

## Discussion

**Figure 4:**
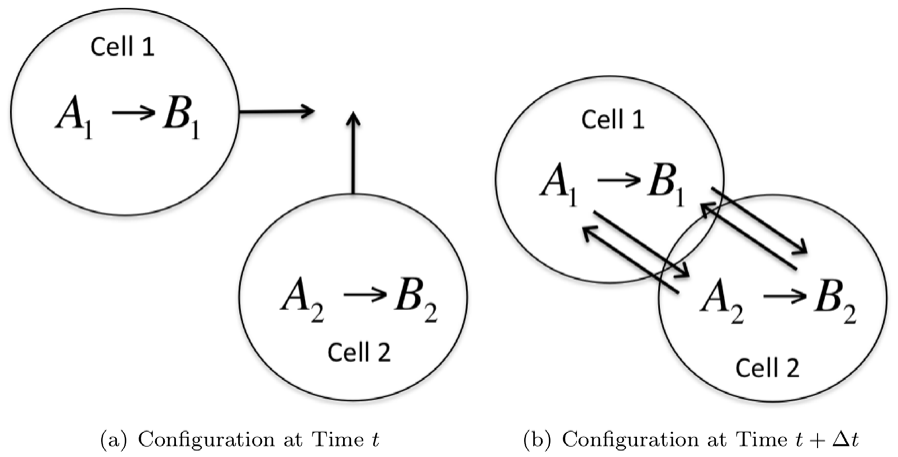
Multi-scale modeling of cells. In this simulation there are two cells. Each has two types of reactants of species type *A, B*. Internally there is a single reaction 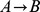 for each cell. Figure 4a shows the configuration at time 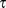 when there is no physical overlap (therefore no interaction) between the cells. The size of the total reaction network is 2. Figure 4b shows overlap between the cells. This sets up diffusion (due to cell membrane paths that may open) of species between the two cells, thus increasing the size of the total reaction network to 6. This sets up a problem where there is a dynamic rearrangement of the total chemical network based on cell configuration.

In this paper we describe a data-parallel implementation of the 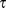-Leaping Method for parallel execution on GPUs. We have obtained an order of magnitude performance gain over the StochKit serial implementation. However, these performance gains are evident only in the regime of very large networks with over 10^5^ reaction channels. Such large systems can occur in two types of scenarios. The first is when the simulation includes a spatial component. Such simulations are typically reaction-diffusion systems where space is discretized into cells and diffusion of species between cells is modeled as a reaction. The fundamental characteristic of such systems is that the basic dynamics within each cell and its interactions with its neighbours are identical for all cells. Using the 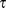-Leaping Method, the whole system can be treated as one large network. However, we believe that the implementation of the Gillespie Multiparticle Method (GMP) on GPUs by Vigelius et al. [19] is a more efficient approach for small networks if it is possible to fit the cell network within the working memory of a single GPU thread. In such situations, it is feasible to use shared memory, thus reducing a significant memory overhead compared with reading from global memory of the GPU. Of course, since this memory is small, the networks simulated are quite small as well. For networks that cannot fit in the memory of a single thread, the implementation by Vigelius will not work. On the other hand, the restriction on our implementation is the total size of the global memory that is quite large (1.5GB is not uncommon on current generation GPUs). The second scenario we envision occurs in multi-scale modeling cell colonies. If these cells are mobile, then based on their location, their interaction changes. Figure 4 illustrates the case of two cells. The number of neighbours that each cell interacts with would not only create a dynamically changing per cell network, but it is unlikely that such networks would fit within the working memory of a single thread. This scenario will require that we treat the entire system as a single network. Of course, such interaction will entail rebuilding the stoichometric data structure based on the spatial configuration of the cells at each update step. However, given our data structure, it is possible to do this in parallel on the GPU. This type of modeling is a topic for future research that will be built on top of our current implementation.

Another type of acceleration that we will investigate in the near future is a combined parallelism across simulations and parallelism within a simulation. As with all stochastic simulations, we have to execute multiple runs to generate dense data sets for analysis. For medium-sized networks, we can assign a thread block to a single run. The low level parallelization will therefore be done at the thread block level with the computation of a single run being distributed across all threads in the thread block. At the same time, multiple thread blocks running concurrently on the GPU can execute multiple runs of the same simulation. Since the stoichiometric matrix is common across all simulations, a single copy will be held either in global or constant memory (depending on the network size).

## Methods

The GSSA assumes a well-stirred system (spatially homogeneous) of *M* molecular species 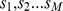 and *N* reaction channels 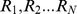, in a fixed volume, at a constant temperature. The system evolves over time with one or more reaction channels being applied to the system at each time step. The state of the system is given by 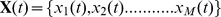, where 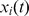 is the number of molecules of 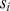. Each reaction channel 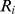, has a reaction propensity 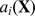 and an associated state change vector 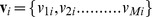, where 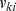 is the change in the number of molecules of species 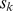 if the reaction channel 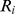 is fired once. Given 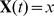, the quantity 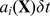 gives the probability that reaction 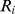 will occur once in the next infinitesimal time interval 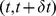.

In the DM, the system advances by firing one reaction at a time. The reaction µ to be fired next is given by the equation:
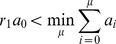
(1)The time increment 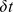 is given by:
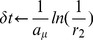
(2)where 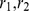 are uniform random numbers and 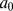 is the sum of all propensities. Finally, the state is updated as:



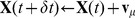
(3)Because DM advances one reaction at a time, it is not very scalable. The 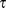-Leaping Method [20] addresses the scalability by processing multiple reactions in a given step. It assumes a certain amount of de-coupling between reactions determined by the leap condition that bounds the relative changes in reactant populations in the given time 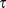 The leap condition is given by:
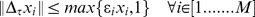
(4)The values 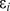 are chosen such that changes in propensity functions are at least bounded by 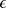. The values 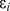 are given by:
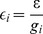
(5)The values 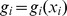 are given by:
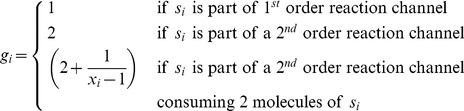
(6)Given the leap condition, the state update is given by:
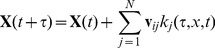
(7)where 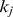 is the number of times a reaction j is fired. It can be approximated by the poisson random variable 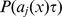 with the expected number of occurrences given by 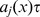.

The selection of 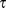 compatible with the Leap Condition is governed by the formula:
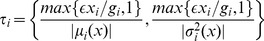
(8)

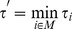
(9)The parameters 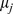 and 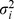 are given by the formulae:
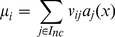
(10)

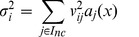
(11)


In these equations 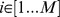, where *M* is the total number of reactants and 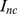 is the set of non-critical reactions. To avoid negative populations due to excessive firing of reaction channels, reactions are classified as critical and non-critical reactions. Critical reactions are defined as those that do not have enough molecular count in reactants to handle 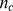 firings. Typically 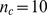. They are simulated using an adapted version of the DM.

### Graphics Processing Units

Originally built for speeding up graphics computation, GPUs have evolved over the years into powerful processors enabling the democratization of high performance scientific computing [21]. GPU vendors have developed application protocol interfaces (APIs) to ease programming efforts [22], [23]. All elements necessary for scientific computing, such as error correction code, support for double precision, etc., are available on the latest generation GPUs.

**Figure 5:**
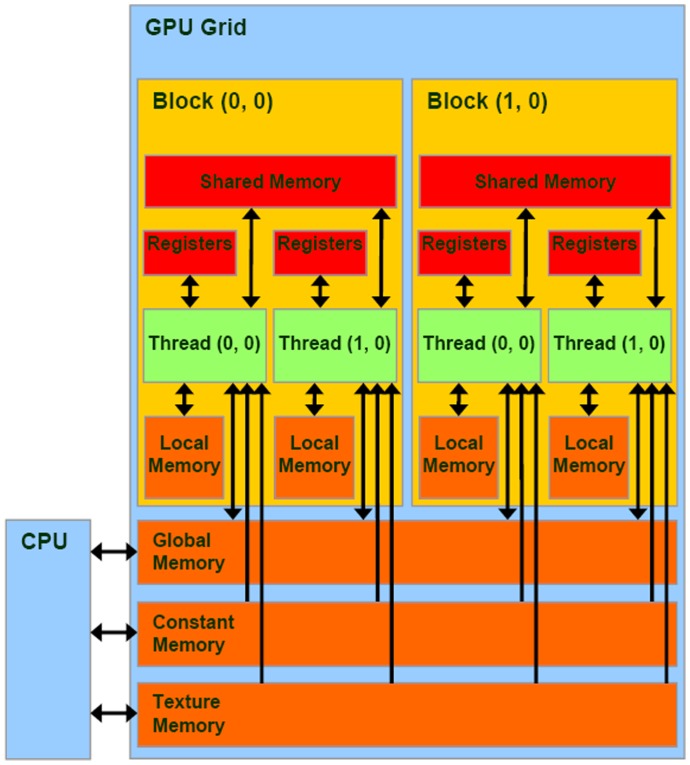
CUDA computing model.

The basic execution unit is a thread. Threads are grouped into thread blocks. Threads in a thread block can communicate with each other because they share a user-controlled cache called shared memory. At the hardware level, threads are grouped into warps. All threads in a warp execute in lock step, i.e., the same instruction at the same time. The program that is executed by every thread in a single parallel invocation is called a *kernel*. Figure 5 illustrates the computing model for NVIDIA GPUs. There are four different types of memory: constant memory - is used for data that is static over the life of the simulation, global memory - is equivalent to CPU random access memory, shared memory - is equivalent to CPU cache but is user controlled, and registers. Algorithms and data-structures have to be designed to match this computational architecture. Moreover, there must be enough parallelism to fully use all computational resources on the GPU. Several textbooks provide an excellent overview of GPU architectures and the related programming models/paradigms [24], [25].

We make extensive use of the Thrust library from NVIDIA for our parallelization [26]. In particular, we use efficient implementations of the generic parallel reduction and scan algorithms combined with transform iterators. Transform iterators are special iterators that take in a vector of data elements and apply a user-defined transform to each element in the input vector. The user-defined transforms are programmed by using functors. For example, given a vector 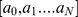, we can find the sum 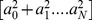 in a single kernel call by using a transform functor that transforms each element as 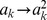 and then using a reduction operation on the transformed entries.

### Implementation Details

**Figure 6:**
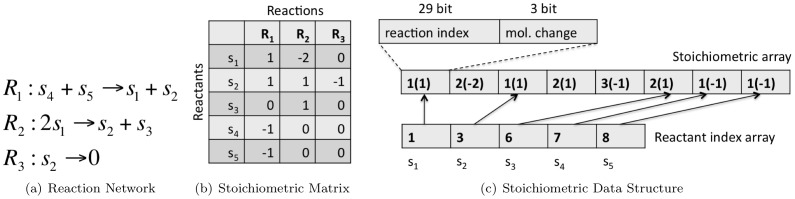
GPU data-structure for stochiometric matrix.

The data structures we use in our implementation can be divided into three groups, namely, stoichiometry data, reactant data, and reaction data. The stoichiometry data represents the matrix 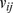, 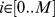, 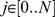. The rows of this matrix indicate reactants, and the columns indicate reactions. This matrix is very sparse, with each column having at most four entries. This is because, at most, each reaction can only affect four reactants. Furthermore, the values 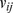 can only be one of 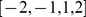. We use a linear array to store the stoichiometric information. Each element of the stoichiometric array is 32 bits wide, with the first 29 bits indicating the reaction index and the next three bytes indicating the change in molecular count. An additional array of indices stores the start index into the stoichiometric array for each reactant. Figure 6 illustrates a case with three reactions 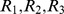 and 5 species 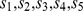. This data structure enables parallel access of data on a per reactant basis both for updating the molecular count, as in equations 3,7, and for computing µ and 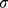, as in equations 10, 11.

The reaction data consist of an array of integers (**X**) that hold the molecular counts of various reactants (system state) and an array of integers (**o**) that classifies the reactant based on its higher order reaction (HOR). The reaction data consist of the following 10 arrays: an array (**t**) of integers that classify the reactions (as uni-molecular, bi-molecular, and bi-molecular with a single reactant), an array of boolean values (**Q**) that classifies reactions as critical/non-critical, an array of integers indicating the index of the first reactant (**s0**), an array of integers indicating the index of the second reactant (**s1**), an array of floats to hold the reaction rate constants (**r**), an array of floats (**a**), that holds the computed reaction propensities, an array of floats(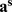) that holds the partial sum of propensities, an array of floats (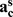) that holds the partial sum of propensities of critical reactions, an array of integers (**k**) that holds the number of times each reaction is fired, and finally, an array of unit4 that holds the state for the random number generator for each reaction.

All data are initialized on the CPU and transferred to the GPU in the beginning. To enable improved cache hit rates on the latest GPUs, we sort the list of reactions using the reactant indices as key. This places reactions sharing reactants near each other in memory. Depending on the specifications in the input file, either the entire trajectory of certain reactants over the total simulation time or the final state of the system can be retrieved at the end of the execution. Data collection and processing is done on the GPU itself to avoid costly CPU-GPU data transfer. As with most GPU parallelizations, the CPU does very little computation and mostly manages the overall execution.

Algorithm 1 (Figure S1) illustrates the general flow of the 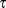-Leaping method. It is clear that classifying reactions as critical and non-critical reactions, computing propensities for each reaction are per reaction computations that can be performed in parallel (lines 7–8 in algorithm 1). A reaction 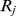 is labeled critical if the molecular count of any reactant 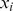 satisfied 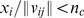. Also, computing the sum of propensities is a reduction operation with the length equal to the number of reactions *N* and uses the results from the previous two steps (line 9 in algorithm 1). We combined these three operations into a single call to the parallel scan algorithm. The transform functor, illustrated in algorithm 2 (Figure S2), acts on the reaction data arrays, scans the reactants’ data array using indices from the reaction data array, and, classifies the reactions as well as computes the propensities. The computed propensities are used by the inclusive scan algorithm to compute the partial sums of all reaction propensities, as well as the sum of all critical reaction propensities in the same call.

Computing the time leap 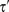 involves a per reactant computation of 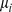 and 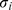 followed by finding the minimum of 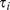 among all reactants. The latter is a reduction operation. The transform functor (illustrated in algorithm 3 (Figure S3)) in this case reads the stoichiometric data structure to find the reactions in which a particular reactant participates. It then computes 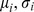 as in equations 10 and 11. Furthermore, the functor computes 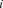, 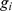 and finally 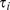 as in equations 6, 5, 8 respectively. The resulting 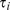 are used by the reduction algorithm to finally compute 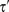 and in equation 9.

The algorithm requires computing the DM, in certain cases, over the entire set of reactions (lines 12–14 of algorithm 1), and in other cases, only over the set of critical reactions (lines 16 of algorithm 1). In the case of running the DM over the entire set of reactions, we have implemented a GPU-based parallel version of the Optimized Direct Method [5], which is illustrated in algorithm 4 (Figure S4). In the case of executing DM on critical reactions, we already have the partial sums of the critical reaction in 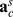. We only execute lines 3,5 from algorithm 4. The 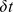 calculated in line 5 is 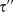 from line 16 in algorithm 1.

The next step in the algorithm is to calculate the number of times 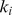 each reaction is fired within the time leap 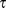. There are two cases here. One sets 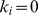 for all critical reactions. The other sets 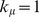 for one critical reaction (the reaction to be fired from line 23 of algorithm 1) and sets 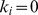 for all other critical reactions. We use a single kernel with an input parameter 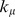 that computes the two cases. For non-critical reactions, 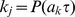. Here 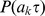 is a poisson random number. For large 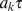 (in our implementation we use the limit 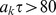), the Poisson distribution is well approximated by a normal distribution with mean 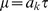 and standard deviation 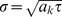. Each thread handling a reaction implements a serial Poisson RNG. In the case of the normal distribution approximation, we use the Box-Muller transform to generate the normal random number from a uniform random number. Each thread runs its own uniform random number generator (URNG). We use the combination Taustworthe-LCG URNG that has the advantage of speed as well as a small state vector and a relatively large period. Each of the Taustworthe-LCG URNG streams has four 32-bit state values that give a period of 2^121^. If the three Tausworthe states are greater than 128, and all four states are initialized using a separate random number generator, each stream can generate up to 2^64^ reasonably uncorrelated random numbers [27]. This is more than sufficient for the purposes of the 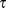-Leaping Simulation. The small state of the URNG means that we can effectively hold it in a thread’s registers and generate an unspecified number of RNs without writing the state back to global memory. We use a Mersenne Twister RNG (MTRNG) implementation on the CPU to seed the Taustworthe-LCG URNGs on the GPU.

The final step is to update the state vector **X**(*t*). One possible option was to use the optimized sparse matrix multiply available from CUBLAS [28]. However, we found that our algorithm has a performance advantage of 50-70% because of the structure and nature of the data. Since the molecule count of each reactant is independent, this step can be parallelized on a per reactant basis. We once again use our stochiometric matrix to accomplish this step. A single thread is assigned to each reactant. The thread reads the stoichiometric matrix to find the reactions that involve this reactant and the related change in molecular count. It also reads the number of times a given reaction is fired from the **k** array. It then updates the molecular count for that particular reactant. Algorithm 5 (Figure S5) illustrates this procedure.

## Supporting Information

Figure S1

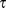
 **-Leaping Method.**
(TIFF)

Figure S2 **Functor for computing propensities.**
(TIFF)

Figure S3
**Functor for computing 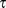-leaping time step.**
(TIFF)

Figure S4 **Parallel Optimized Direct Method.**
(TIFF)

Figure S5 **Functor for updating molecular count of reactants.**
(TIFF)

Appendix S1 **Generating Random Consistent Synthetic Networks.**
(PDF)
