# Supplementary figures and images for: Accelerating the Gillespie τ-Leaping Method Using Graphics Processing Units
Source: PLoS One. 2012 Jun 8;7(6):e37370. doi: 10.1371/journal.pone.0037370 (PMC3371023; doi:10.1371/journal.pone.0037370)

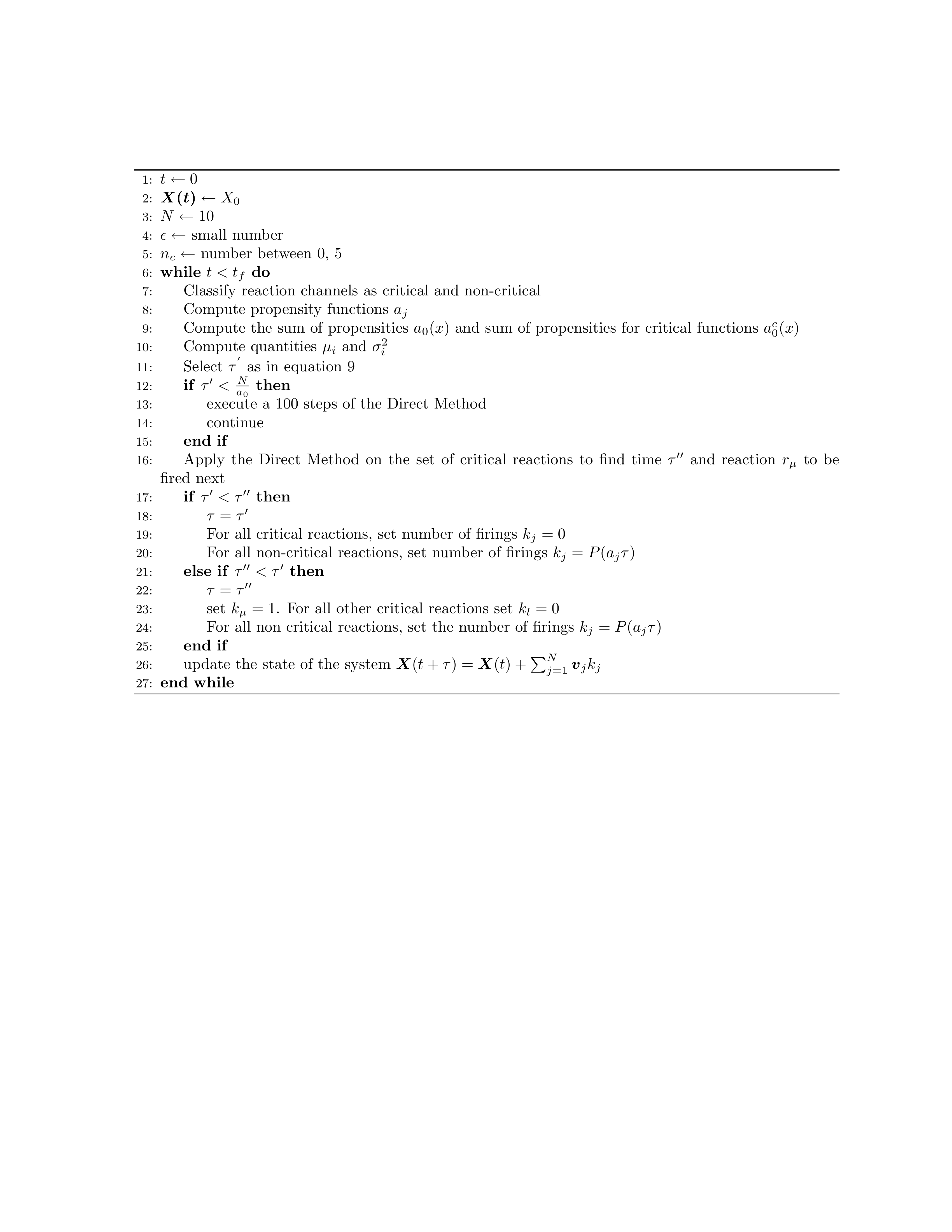

Supplement: Figure S1 — -Leaping Method. (TIFF) [file pone.0037370.s001.tif]

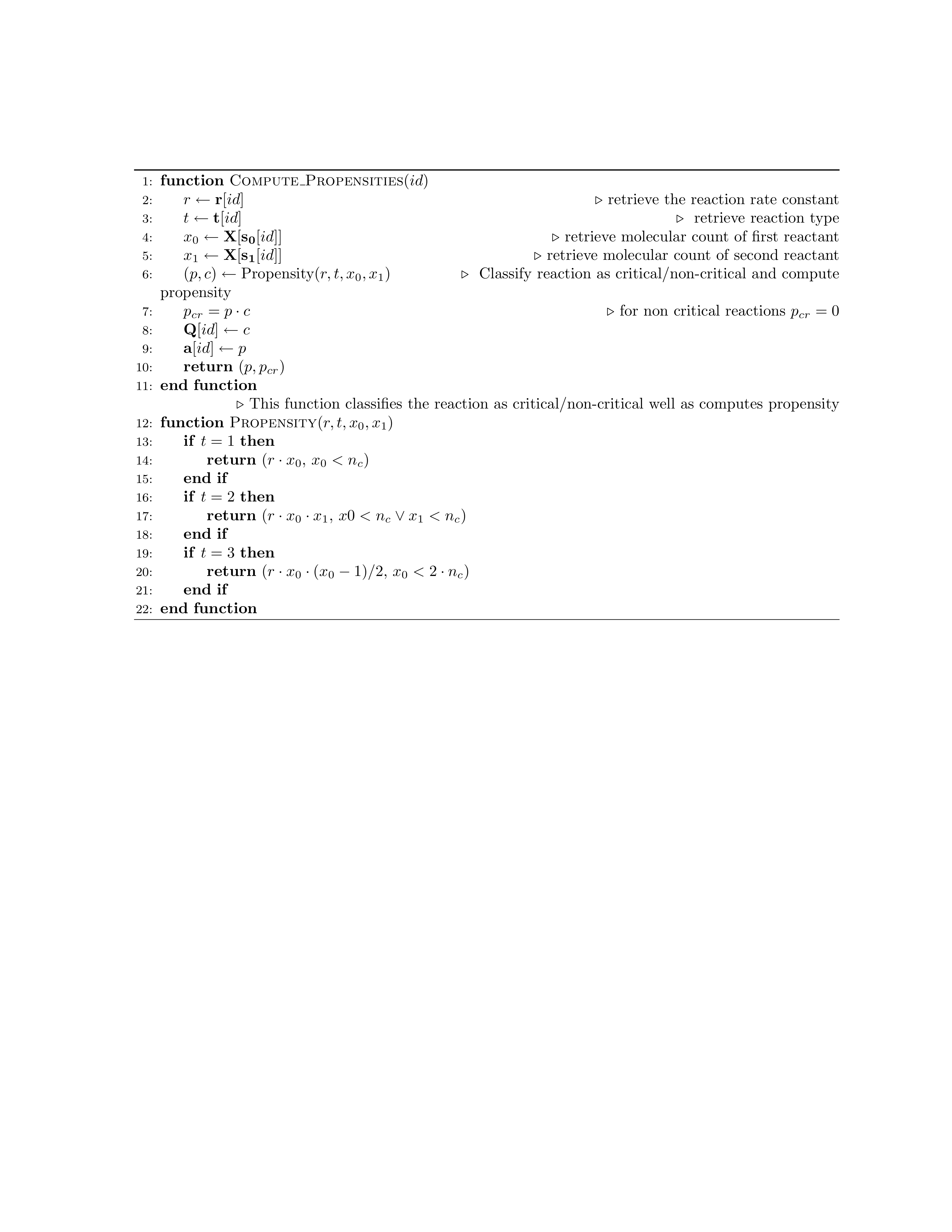

Supplement: Figure S2 — Functor for computing propensities. (TIFF) [file pone.0037370.s002.tif]

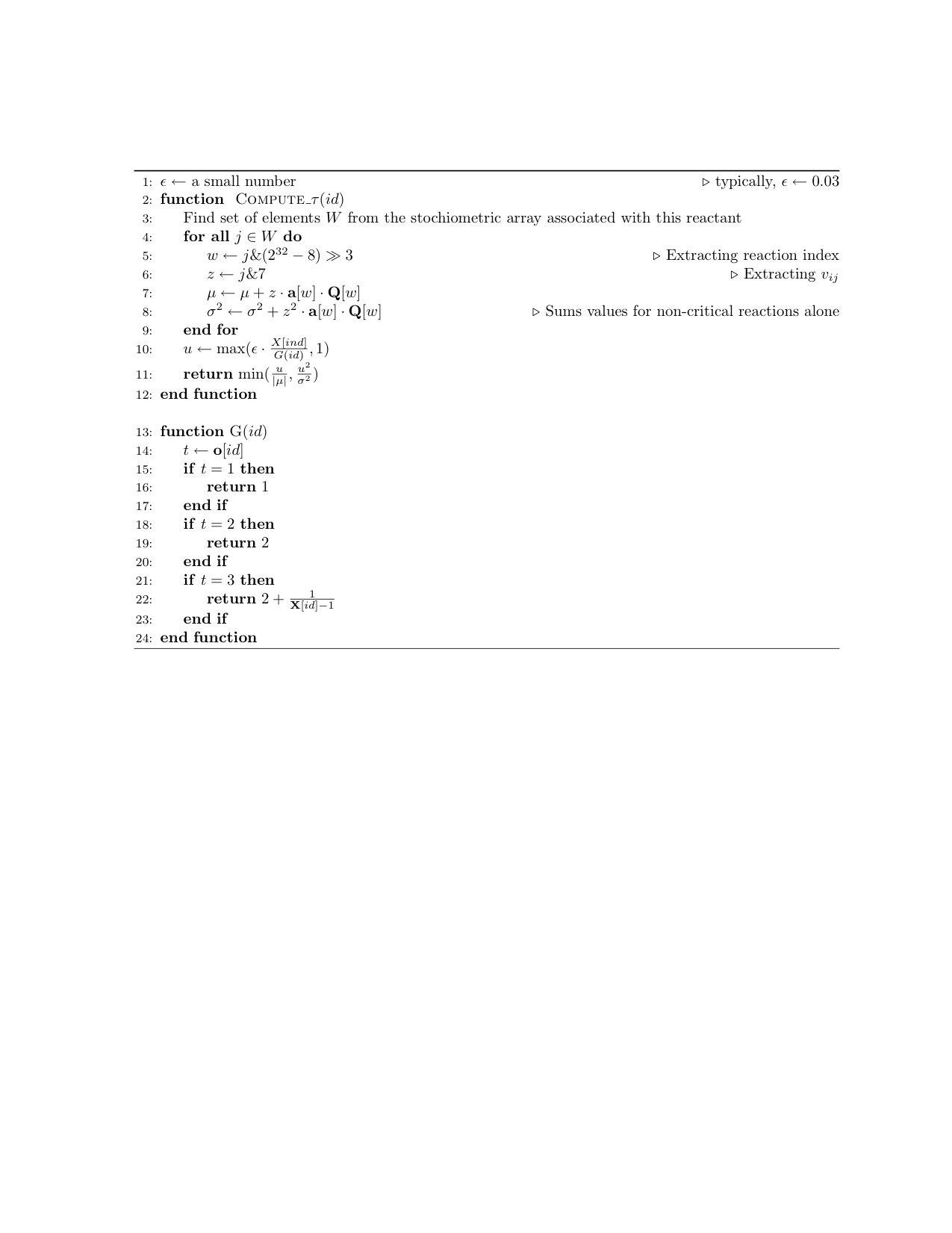

Supplement: Figure S3 — Functor for computing -leaping time step. (TIFF) [file pone.0037370.s003.tif]

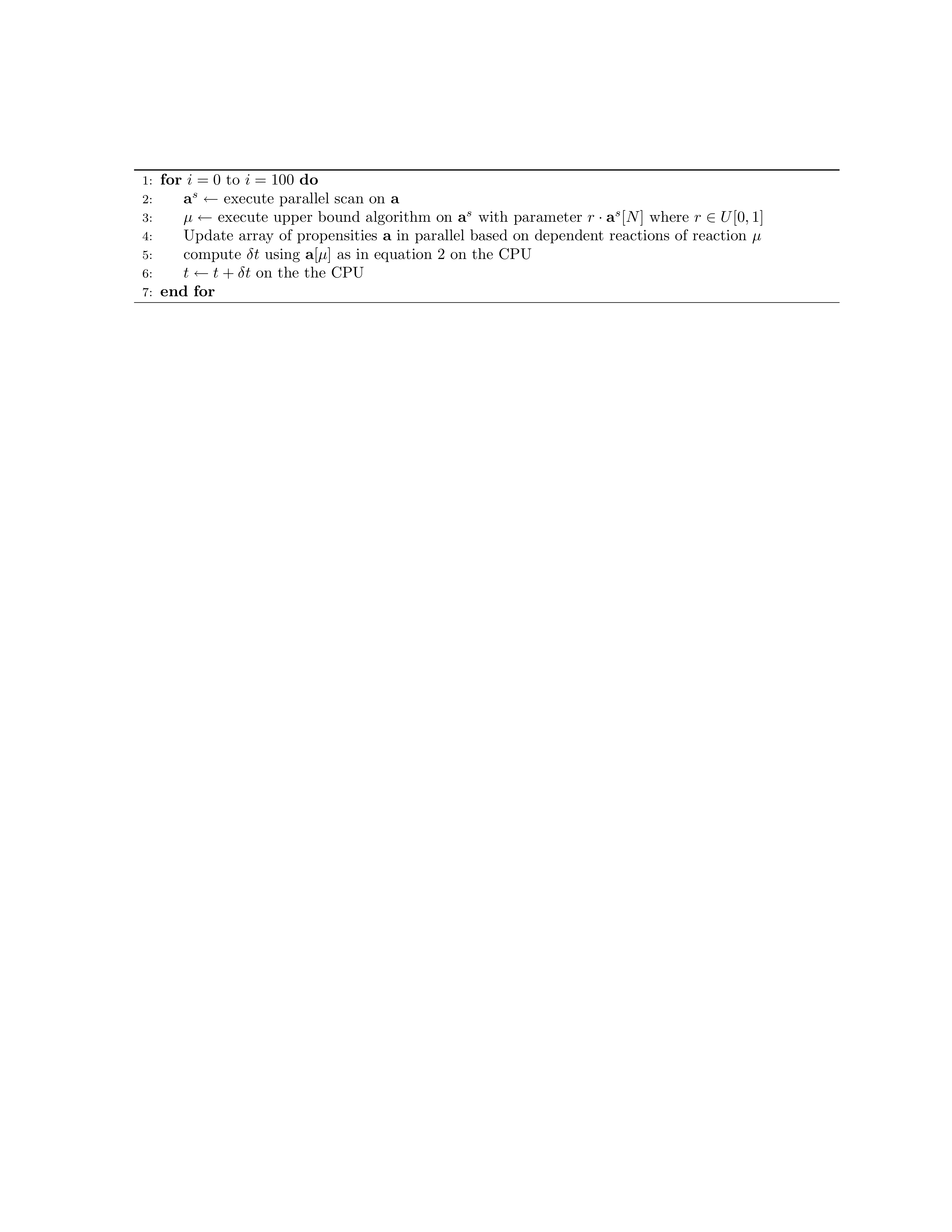

Supplement: Figure S4 — Parallel Optimized Direct Method. (TIFF) [file pone.0037370.s004.tif]

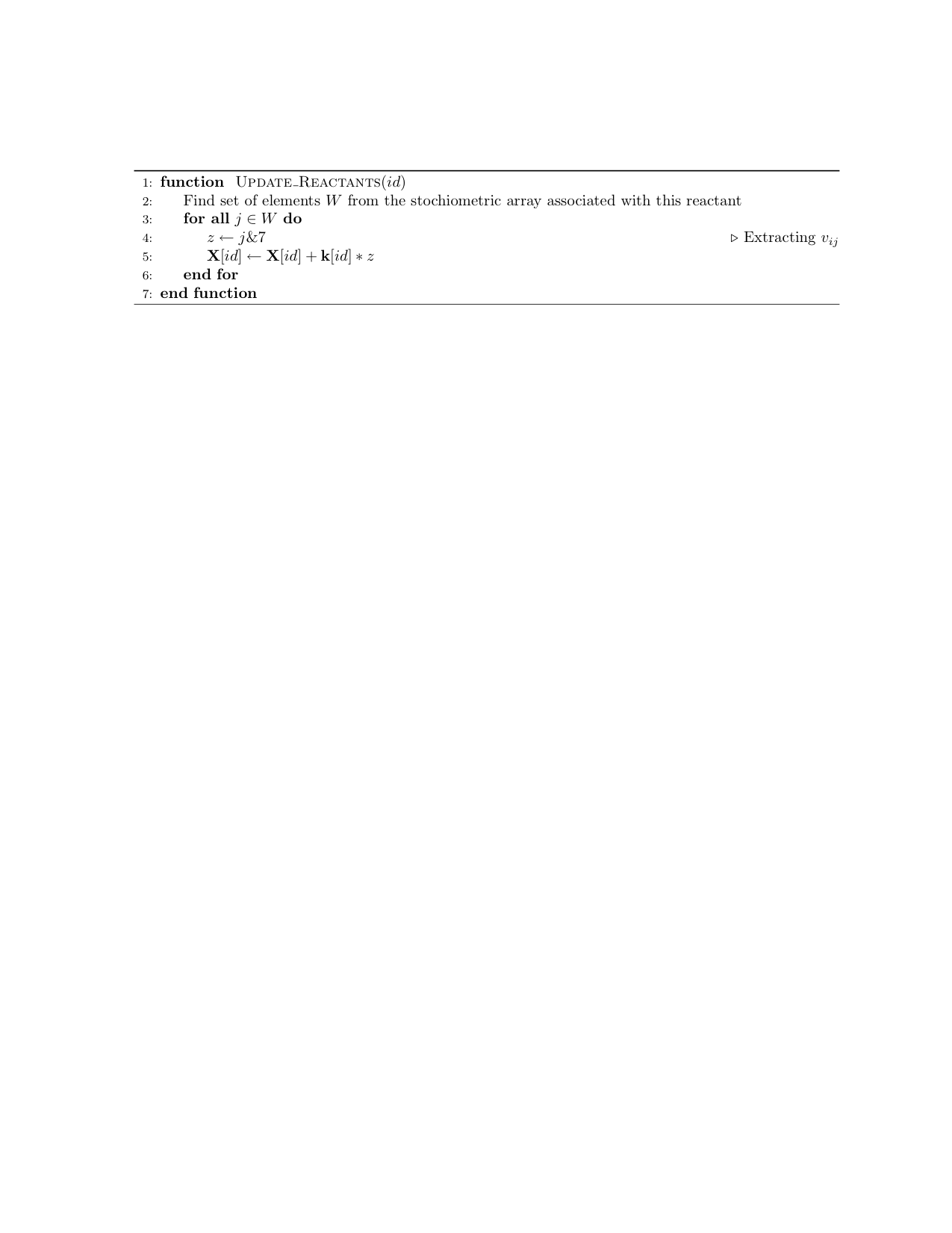

Supplement: Figure S5 — Functor for updating molecular count of reactants. (TIFF) [file pone.0037370.s005.tif]
