# Supplementary material for: Accelerating the Gillespie τ-Leaping Method Using Graphics Processing Units
Source: PLoS One. 2012 Jun 8;7(6):e37370. doi: 10.1371/journal.pone.0037370 (PMC3371023; doi:10.1371/journal.pone.0037370)
Supplement: Appendix S1 — Generating Random Consistent Synthetic Networks. (PDF) [file pone.0037370.s006.pdf]

Appendix S1: Generating Random Consistent Synthetic Networks

Zero order	$\phi \rightarrow S_i$
First Order	$S_i \rightarrow \phi$<br>$S_i \rightarrow 2S_k$<br>$S_i \rightarrow S_k + S_l$
Second Order	$S_i + S_j \rightarrow \phi$<br>$S_i + S_j \rightarrow S_k$<br>$S_i + S_j \rightarrow S_k + S_l$
Special Second Order	$2S_i \rightarrow \phi$<br>$2S_i \rightarrow S_k$<br>$2S_i \rightarrow S_k + S_l$

Table 1. All possible reaction types

To evaluate our algorithms we use random consistent synthetic networks. These synthetic networks can have reactions of the types shown in Table 1. The input to our network generator is the number of reactants N, the number of each of the different types of reactions, and the total number of reactions M. We randomly select the reaction type (zero order, first order, second order, or special second order) and subtype to be generated at each step. Furthermore, we randomly select the reactants for a given reaction. We maintain a hash table of all reactions that are generated to make sure that reactions are not duplicated. The hash is basically a permutation of the two reactants and two product indices. For example, a second order reaction $S_i + S_j \rightarrow S_k + S_l$, will have the hash $[i, j, k, l]$ which is the same as $[i, j, l, k]$, $[j, i, l, k]$, and $[j, i, k, l]$. A first order reaction $S_i \rightarrow S_k + S_l$ will have the hash $[i, -1, k, l]$, which is the same as $[i, -1, l, k]$, $[-1, i, l, k]$, and $[-1, i, k, l]$. If there are less than two reactants or two products, then the corresponding index in the hash is set to -1. The reaction rates are randomly selected as $k_r \leftarrow U[0, 1]$ for each reaction.
